# Malignant fibrous histiocytoma, now referred to as Undifferentiated Pleomorphic Sarcoma: A Case Report of an unexpected histology of a subcutaneous lesion

**DOI:** 10.1016/j.ijscr.2019.06.035

**Published:** 2019-06-22

**Authors:** Karishma Seomangal, Nezar Mahmoud, Joseph P. McGrath

**Affiliations:** Department of General Surgery, Our Lady’s Hospital, Navan, Co. Meath, Ireland

**Keywords:** Case report, Malignant fibrous histiocytoma, Undifferentiated pleomorphic, Sarcoma, Storiform, Myxoid, Subcutaneous mass

## Abstract

**Introduction:**

An 85 year old male presented to his General Practitioner with a vague history of a lump on his back that was mobile and non tender. He had a previous history of advanced prostate cancer and radiotherapy treatment.

**Presentation:**

We present an unexpected diagnosis of malignant fibrous histiocytoma of a subcutaneous lesion removed from the patient’s back.

**Discussion:**

Excisions of subcutaneous lesions along with cutaneous lesions form the majority of cases on a usual minor operations list. All lesions are sent for histopathological analysis and most are confirmed benign sebaceous cysts or lipomas. However the entity of malignant fibrous histiocytoma, now referred to as undifferentiated pleomorphic sarcoma (UPS) should be kept in mind.

Patients will usually present late with advanced local disease or metastasis, usually to lungs and lymph nodes. Treatment is surgical with wide local excision and neoadjuvant/adjuvant radiotherapy has been advocated as well.

**Conclusion:**

Undifferentiated pleomorphic sarcoma should be a differential in subcutaneous lesions in older adults with a previous history of malignancy, radiation therapy and a mass in the subcutaneous tissue of the back.

## Highlights

• Think of undifferentiated pleoimorphic sarcoma as a differential in older adults with a history of radiation therapy.
• It is important to discover the type and grade of the tumor.
• Patients should be thoroughly worked up for the best possible outcome.

## 1 Introduction

Malignant fibrous histiocytoma is a neoplasm believed to originate from primitive mesenchymal cells; arising from soft tissue or bone, usually in the extremities or retroperitoneum [1]. It is now referred to as undifferentiated pleomorphic sarcoma (UPS) and is classified under the undifferentiated/unclassified sarcomas group [2,3]. This neoplasm is the most common soft tissue sarcoma of late adult life [4] and has a slight male predominance. It has been categorised into five subtypes: (a) storiform/pleomorphic, (b) myxoid, (c) giant cell, (d) inflammatory and (e) angiomatoid. The most common is storiform/pleomorphic that forms 50–60% of all such tumours while myxoid type is the second most common at 25% [5]. One risk factor is radiotherapy [[6], [7], [8]]; indeed about 20% of all sarcomas are the undifferentiated/unclassified type and about a quarter of those are radiation related [3]. Patients usually present late with metastasis, most frequently to lungs or lymph nodes [4,9]. Imaging like Computed Tomography (CT) or Magnetic Resonance Imaging (MRI) scans play a role in defining the extent of the disease and the treatment is mainly surgical. There may be a role for neoadjuvant/adjuvant radiotherapy [10]. This work has been reported in line with the SCARE criteria [11].

## 2 Presentation of case

**Fig. 1:**
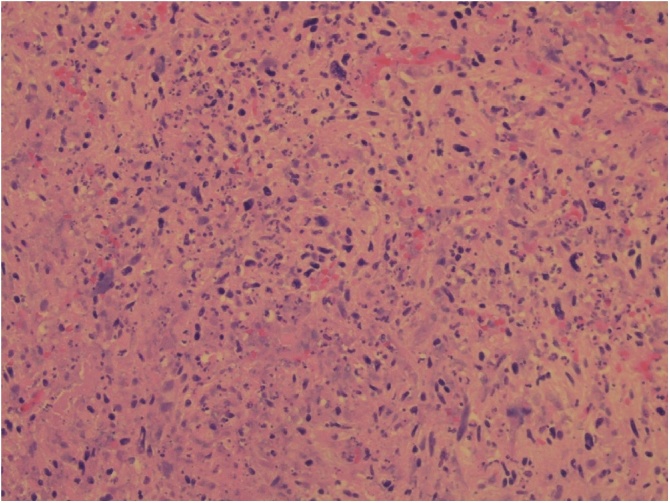
Haematoxylin eosin stain showing necrosis.

**Fig. 2:**
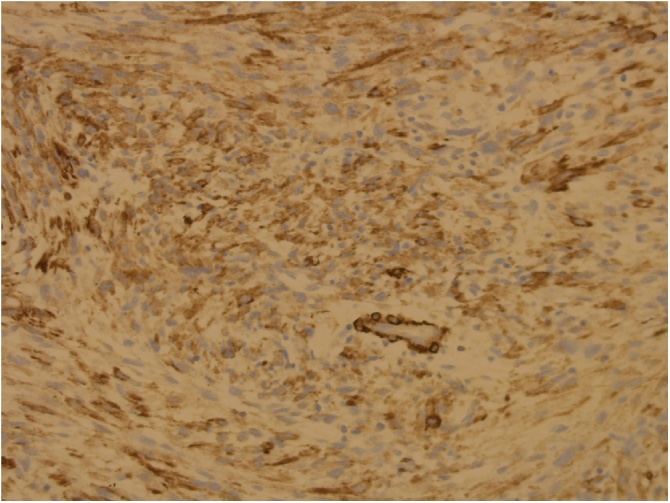
Staining for smooth muscle actin.

An 85 year old male was referred by his General Practitioner with a mobile, non tender lump on his back for excision under our surgical service. He had not had a similar lesion on any other part of his body before. Medical history included active prostate cancer, mixed hyperlipidaemia, hypertension, NSTEMI, TIA, paroxysmal atrial fibrillation and gastritis/duodenitis. Current medication included Aspirin, Omnic, Rosuvastatin, Furosemide, Folic acid, Omeprazole, Lexapro and Ensure Plus. He had been on Casodex (antiandrogen) and received radiotherapy treatment for prostate cancer. On physical examination the lump was mobile and non tender; no lymphadenopathy appreciated. On operation the lesion was discovered to be deep, extending down to the fascia involving muscle and extensively vascular. Two pieces measuring 45 mm × 35 mm × 22 mm and 35 mm × 15 mm × 15 mm was removed in entirety with clinically adequate margins having its malignant potential recognised. The histology showed it to be a malignant fibrous histiocytoma (undifferentiated sarcoma), pleomorphic type, Grade 3 (Fig. 1, Fig. 2). Unfortunately residue tissue was still present at the margins.

**Fig. 3:**
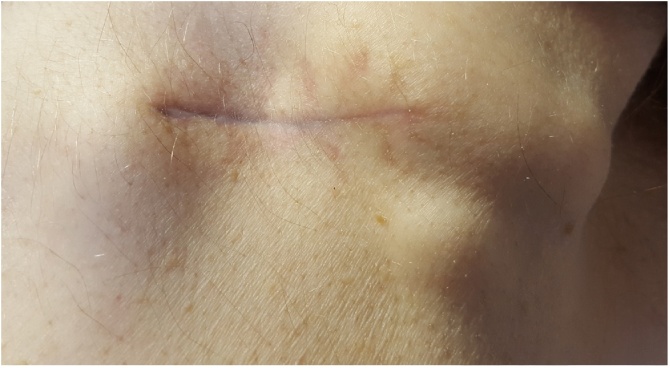
Recurrence of tumour at ends of healed wound.

**Fig. 4:**
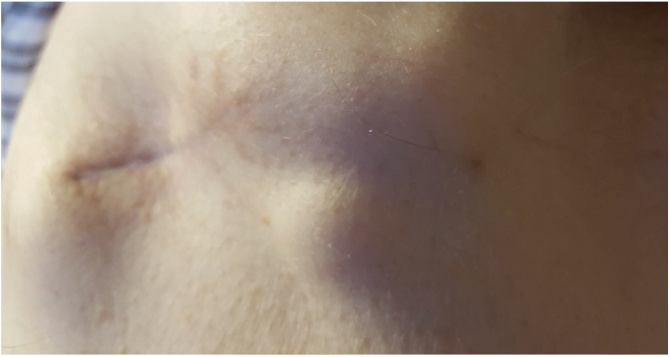
Tumour recurrence at ends of healed wound.

Follow up was 2 weeks post procedure in OPD with wound healing well. He had a CT TAP which showed no supraclavicular or axillary lymph adenopathy and sclerotic lesions in the skeleton consistent with metastases of his prostate cancer. Previous CT brain and CXR was clear. Referral to an Oncologist was made and treatment was radiotherapy. On the next Surgical OPD appointment at 4 months the wound had healed well with new growths at the limits of the excision (Fig. 3, Fig. 4). The plan was to continue the radiotherapy and follow in OPD. This patient had several admissions to hospital with dyspnoea and exacerbation of CCF and succumbed to respiratory complications.

## 3 Discussion

Malignant fibrous histiocytoma, now referred to as undifferentiated pleomorphic sarcoma, is the most common soft tissue sarcoma of late adult life [4]. UPS can occur throughout the body with cases of visceral involvement being published [12,16]. Patients are usually between 32–80 years old with a slight male predominance [5]. Our patient’s histology showed the storiform/pleomorphic subtype that forms 50–60% of all such tumours while myxoid type is the second most common at 25% [5]. The other forms are rarer. Interestingly, the inflammatory subtype mainly occurs in the retroperitoneum [3].

Symptoms are usually a painless, enlarging palpable mass [5], like the case with our patient. Local mass effect symptoms may be caused depending on location.

Risk factors can include radiation treatment for another malignancy like Hodgkin’s lymphoma [7], post breast cancer resection radiotherapy [8], background history of Paget’s disease, non ossifying fibroma and fibrous dysplasia. Soft tissue sarcoma has been linked to certain syndromes such as Werner, Gardner, Li Fraumeni and Von Recklinghausen [13]. In this case, the major contributing factor to developing UPS was most probably our patient’s previous radiation therapy and his immunosuppressed state with active malignancy that allowed the sarcoma to grow unhindered.

The diagnosis at histology involves microscopy, molecular studies and immunohistochemistry techniques. It is necessary for pathologists to have a consensus in classifying, grading and staging neoplasms and the *World Health Organisation Classification of Tumors of Soft Tissue and Bone* facilitates that [3]. Our patient’s tissue showed a storiform/pleomorphic characteristic and was given a Grade 3 (Fig. 1). The cells showed significant pleomorphism, a high mitotic count of 34/10 HPFs and less than 50% area of necrosis were seen. Immunohistochemical stains were done and the tumour was positive for smooth muscle actin (SMA) (Fig. 2). The tumour was negative for melanocytic markers (HMB-45, mel A), neural markers (S100), desmin, Bcl – 2 and CD34. Further molecular studies include checking for BRAF mutations [13]. BRAF is a gene that codes for *B- Raf*, a proto oncogene that is mutated in many human cancers. Another commonly mutated gene is p16. These mutations, if present, are not limited to malignant fibrous histiocytoma and this type of testing is not routinely done at our laboratory facility.

Imaging has a role with radiographs of the extremity (usual primary site) and chest (usual metastasis) being the first investigation done. Magnetic resonance imaging (MRI) is the most useful test as it gives valuable information about the size, location and proximity to neurovascular structures [3]. Computed Tomography (CT) is an alternative scan and Karki et al. did important work in 2012 correlating CT images and histology retrospectively. It must be emphasised that these imaging investigations do not *diagnose* UPS; a biopsy is needed which is usually excisional if tumors are less than 3 cm. Our patient had the entire lesion removed in two parts which measured 4.5 cm × 3.5 cm×2.2cm and 3.5 cm × 1.5 cm×1.5 cm with clinically adequate margins; the amount of involvement was only determined at surgery hence decision to resect as entirely as possible. Post procedure CT TAP was thus not useful in evaluating the lesion; however confirmed no supraclavicular or axillary lymphadenopathy and sclerotic bony lesions consistent with concomitant prostatic metastases demonstrated. Specialists of choice are Orthopaedic or general surgical oncologists and they may choose additional imaging modalities like bone scans or Positron Emission Tomography (PET) scans.

Staging can be done once biopsy and imaging is completed by using the American Joint Committee on Cancer Staging Manual, 8th edition [2] (AJCCM). These guidelines group soft tissue sarcomas by anatomical site and the histologic subtype, grade and tumor size are essential for staging. TNM categories i.e. tumor size and extent, nodal involvement and distant metastasis are used. Grade is based on the histologic subtype, degree of differentiation, mitotic activity and necrosis and helps determine risk better than the primary tumor size [2]. The grading scale used by the French Federation of Cancer Centers Sarcoma is preferred for its ease of reproducibility and ranges from Gx (cannot be assessed or graded) to Grade 3 (total differentiation, mitotic score and necrosis score is 6, 7 or 8) [2]. Staging is then done using TNM and grading together to range from Stage 1 to Stage 4. Staging for our patient was thus calculated to Stage IIIA (T2 – tumor >5 cm and </= 10 cm in greatest dimension + N0 + M0 + Grade 3).

Prognosis varies and factors include size, grade, location and inflammatory component. Unfortunately most cases are found late at Stage 3 and 4, possibly with metastases. They are aggressive and recur locally [6], which occurred in our patient. In a study done by Kearney et al. [15], a local recurrence rate of 51% was seen in patients with a ‘complete excision’. Pezzi et al. [9] found that the primary tumor size indicated the 5-year survival rate: tumors <5 cm had a survival rate of 82%; 5–10 cm, 68%; and >10 cm, 51%. The intermediate grade tumors showed a 5-year survival rate of 80%, and the 5-year survival rate for high-grade tumors was 60%. Survival rates for both grades were affected by size: tumors of high grade and smaller than 5 cm in diameter had a survival rate of 79%; 5–10 cm, 63%; and more than 10 cm, 41%. Superficial and distal located tumors were better. The size, depth and inflammatory component were important in metastasis; small sized, superficially located or with a prominently inflammatory component metastasized less frequently than large, deeper sited tumors [4,14]. The same staging system is used for the recurrence of lesion indicated with prefix ‘r’. It must be kept in mind that the prognosis for the UPS and prognosis for the prostate cancer in this case had to be assessed jointly.

The main treatment of UPS remains complete surgical excision, with margins of at least 2 cm with a role for neoadjuvant/adjuvant radiotherapy and doxorubicin based chemotherapy [8,10].

## 4 Conclusion

Undifferentiated pleomorphic sarcoma should be a differential of a deeper lying subcutaneous lesion. It frequently presents in an advanced stage in older patients. Past history of radiation therapy is a risk factor. Local, wide resection is advocated with a role for neoadjuvant or adjuvant radiation or chemotherapy. It is very important to grade the tumor and patients should receive a full work up for staging.

## Conflicts of interest

No conflict of interest.

## Sources of funding

Funding by authors.

## Ethical approval

This is a case report submission and ethical approval is not required at our Institution for case reports.

## Consent

Consent has been obtained for the publication of this manuscript and images and is available to the Editor-in-chief of this Journal upon request.

## Author contribution

KS contributed in conceptualization, obtaining data (including consent and images), writing the original draft, editing the final copy and funding.

NM obtained data (including consent and images), contribution to writing and reviewing manuscript and funding.

JPMcG contributed to writing and review of final copy and funding.

## Registration of research studies

Not applicable for a case report.

## Guarantor

KS.

## Provenance and peer review

Not commissioned, externally peer-reviewed.
